# Active Sites of M(IV)-incorporated Zeolites (M = Sn, Ti, Ge, Zr)

**DOI:** 10.1038/s41598-017-16409-y

**Published:** 2017-11-23

**Authors:** Gang Yang, Lijun Zhou

**Affiliations:** 1 College of Resources and Environment, Southwest University, Chongqing, 400715 China; 2 Schuit Institute of Catalysis, Eindhoven University of Technology, Eindhoven, 5600MB The Netherlands

## Abstract

M(IV)-incorporated zeolites have recently aroused wide interest due to outstanding catalytic effects while their active sites remain largely elusive. Here periodic density functional theory calculations are conducted finding that active sites are determined jointly by identity of M(IV) ions, topology of zeolites, type of framework species and choice of T sites. All M2(IV) active sites in BEA zeolites are penta-coordinated with chemisorption of one water while subsequent water molecules that form only H-bonds promote chemisorption of the first water, especially the second water possessing comparable or even higher adsorption strengths as the first water; Ti(IV) and Ge(IV) active sites at the intersection remain penta-coordinated and Sn(IV) and Zr(IV) active sites prefer to hexa-coordination although potentially expanded to hepta-coordination. Different from other zeolites, Ti(IV) active sites in FER zeolites are hexa-coordinated as Sn(IV) active sites, due to the promoting effect of the first water. Lewis acidic defects expand Ti(IV) active sites to hexa-coordination while inhibit the formation of hepta-coordinated Sn(IV) species. Two forms of Brϕnsted acidic defects exist for Sn(IV) sites instead of only one for Ti(IV) sites, and all M(IV) Brϕnsted acidic defects, regardless of different acidic forms and M(IV) ions, can chemisorb only one water.

## Introduction

In the past few decades, M(IV)-incorporated zeolites have gained enough attention^1–3^. The discovery of Titanium silicate-1 (TS-1) in 1983^4^ has been regarded as a milestone in heterogeneous catalysis^5^ and other Ti(IV)-containing zeolites such as Ti-MWW, SSZ-24 and Ti-UTL were subsequently developed that also exhibit satisfying catalytic effects for a wide spectrum of substrates^6–10^. Corma, Přech and their collaborators^11,12^ reported that Sn-BEA zeolite is an efficient catalyst for the Baeyer-Villiger oxidation reactions. As a matter of fact, Sn- and Zr-BEA zeolites are also known to exhibit superior catalytic performances for the transformation of cellulosic biomass to downstream chemicals and fuels^3,13–15^. Albeit less used for catalytic applications, Ge(IV) ions introduced into the framework of zeolites have the directing effect that results in the formation of ITQ-24, ICP-2 and other new structures, and the ADOR (assembly-disassembly-organization-reassembly) mechanism was posed for the synthesis processes^16,17^.

Isolated and well-defined M(IV) sites have been acknowledged as the active sites of M(IV)-incorporated zeolites^1–3,5,13–15,18–20^. As testified by density functional and two-layer ONIOM calculations, M(IV) ions exhibit outstanding adsorption performances for a variety of probe molecules such as H_2_O, NH_3_ and amino acids^21–40^. The Ti-peroxo species in TS-1 zeolite produced from the interaction of Ti(IV) sites and H_2_O_2_ are the active sites for alkene epoxidation^41–57^, and Wells *et al*.^48^ demonstrated that Ti(IV) sites adjacent to Si vacancies [(OSiO_3_)_3_TiOH] are more reactive; that is, the epoxidation processes are significantly accelerated by defects. The two Ti(IV) species [i.e., Ti(OSiO_3_)_4_ and (OSiO_3_)_3_TiOH] were identified in TS-1 zeolite^49,50^ and Sn(IV) analogues were proposed for Sn-BEA zeolite^51–53^. Defects in Sn-BEA zeolite were reported to play a significant promoting effect during the transformation of cellulosic biomass^54–60^.

**Figure 1:**
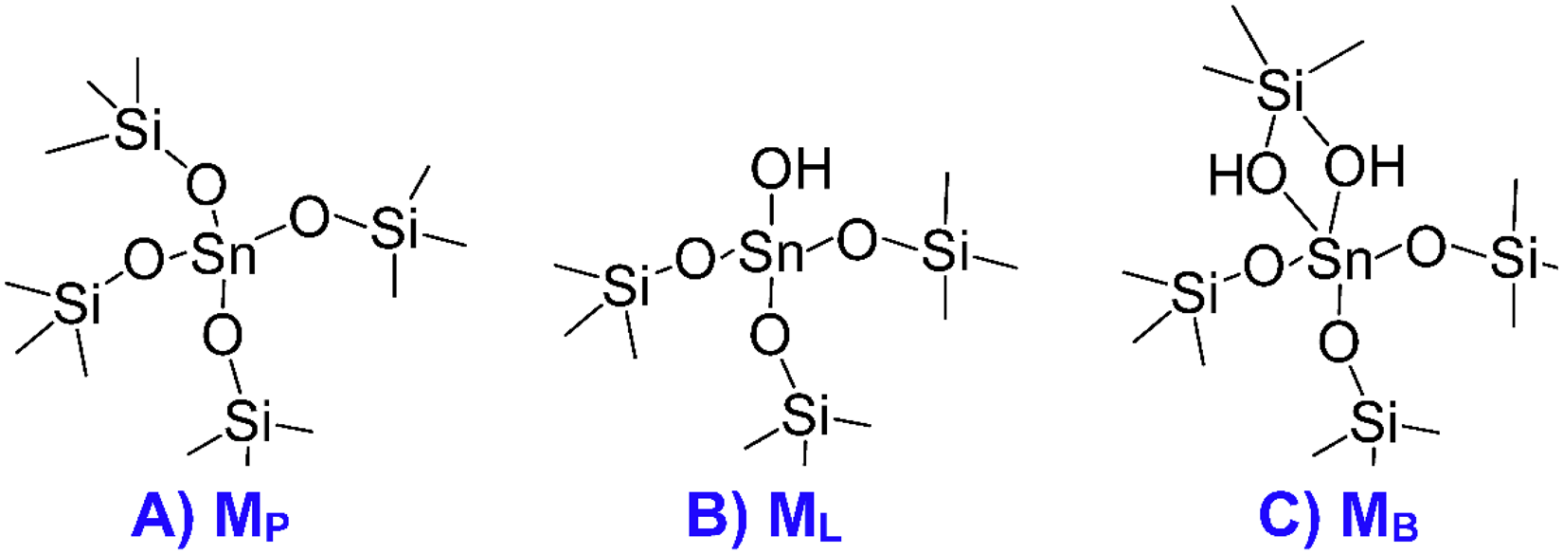
Different framework M(IV) species within zeolites that are referred to as (**A**) perfectly tetrahedral M(IV) sites (**M**_**P**_), (**B**) defects with Lewis acidity (**M**_**L**_) and (**C**) defects with Brϕnsted acidity (**M**_**B**_).

Active sites are one of the central topics for adsorption and catalysis, while a number of key issues remain enigmatic for M(IV)-incorporated zeolites; e.g., we are not clear which causes the differences of active sites in Sn-BEA and TS-1 zeolites, topology of zeolites or identity of M(IV) ions. The active sites of Sn-BEA zeolite were assigned to be octahedral Sn(IV) species composed by tetrahedral Sn(IV) sites and two water molecules^61^, while the Ti(IV) active sites in TS-1 zeolite prefer to being five-coordinated upon water adsorption^62^. In this work, periodic density functional theory (p-DFT) calculations were conducted to have a comprehensive understanding of M(IV) active sites and their coordination numbers in M(IV)-incorporated zeolites, considering the effects such as topology of zeolites (BEA, FER, CHA), identity of M(IV) ions (M = Sn, Ti, Zr, Ge), type of framework species (**M**_**P**_, **M**_**L**_, **M**_**B**_, see Fig. 1) and choice of crystallographically distinct T sites: (1) M2(IV) active sites in BEA zeolites containing the various M(IV) ions (M = Sn, Ti, Zr, Ge). The second water that forms only H-bonds promotes chemisorption of the first water and has comparable or even larger adsorption energies; (2) M9(IV) and M6(IV) active sites in BEA zeolites, where Sn(IV) and Zr(IV) active sites are hexa-coordinated while Ti(IV) and Ge(IV) active sites remain penta-coordinated; (3) M(IV)-BEA zeolites with adsorption of three and four water molecules. The third water at M2(IV) sites continues to promote chemisorption of the first water while subsequent adsorption may play an adverse effect. Sn(IV) and Zr(IV) active sites are potentially expanded to hepta-coordination; (4) M(IV)-CHA and M(IV)-FER zeolites. Different from other zeolites, Ti(IV) active sites in FER zeolites prefer to being hexa-coordinated as Sn(IV) active sites; (5) Lewis acidic defects in M(IV)-BEA zeolites, which produce significantly beneficial effects for water adsorption. The coordination number of Ti(IV) sites is expanded to six while the hepta-coordinated Sn(IV) species is inhibited; (6) Brϕnsted acidic defects, which promote chemisorption for the first water while prevents the second water from chemisorption. A second form of Brϕnsted acidic defects with higher stability was detected in Sn-BEA zeolite that shows distinct adsorption properties. Results obtained thus far are beneficial to understand the structural, adsorption and coordination aspects of M(IV)-incorporated zeolites and to decipher the active sites that are critical to adsorption and catalytic processes.

## Computational Details

### Models

**Figure 2:**
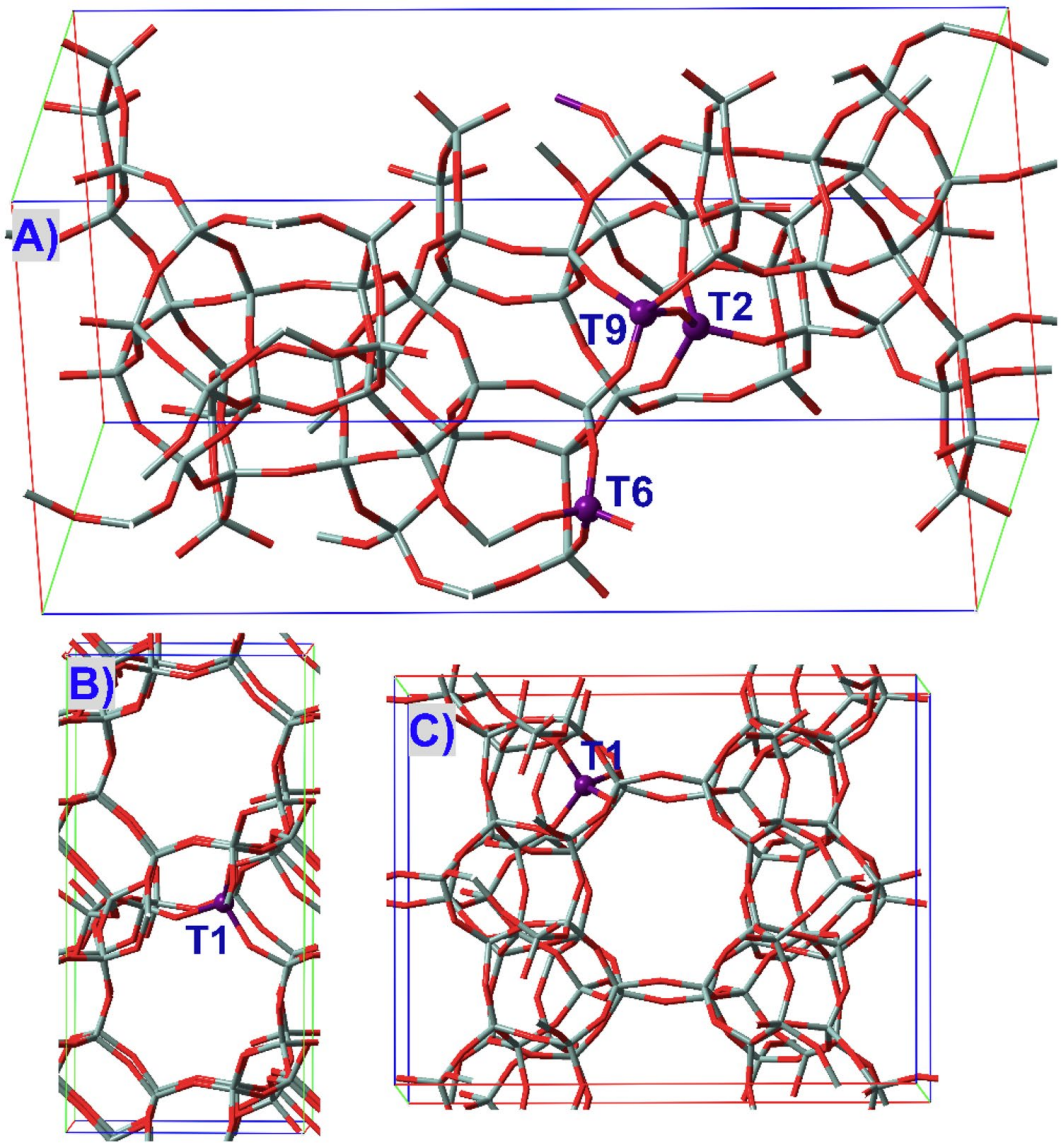
Periodic models of (**A**) BEA, (**B**) CHA and (**C**) FER zeolites labeling the T sites presently investigated.

Atomic coordinates of zeolites were downloaded from the International Zeolite Association (IZA) website^63^. The periodic models of M(IV)-BEA, M(IV)-FER and M(IV)-CHA zeolites as used previously^39^ were displayed in Fig. 2, wherein M(IV)-FER and M(IV)-CHA consist of two and four unit cells along the *c* (1 × 1 × 2) and *a* × *b* (1 × 2 × 2) lattice vectors, respectively. Different from CHA and FER zeolites where all T sites are indistinguishable (referred to as T1), BEA possesses nine crystallographically distinct T sites, and as recommended elsewhere^28–30,33,35,38,39,53–61^, T2 site that is the most energetically favorable and T9 and T6 sites that are situated at the intersection of two channels were investigated.

**Figure 3:**
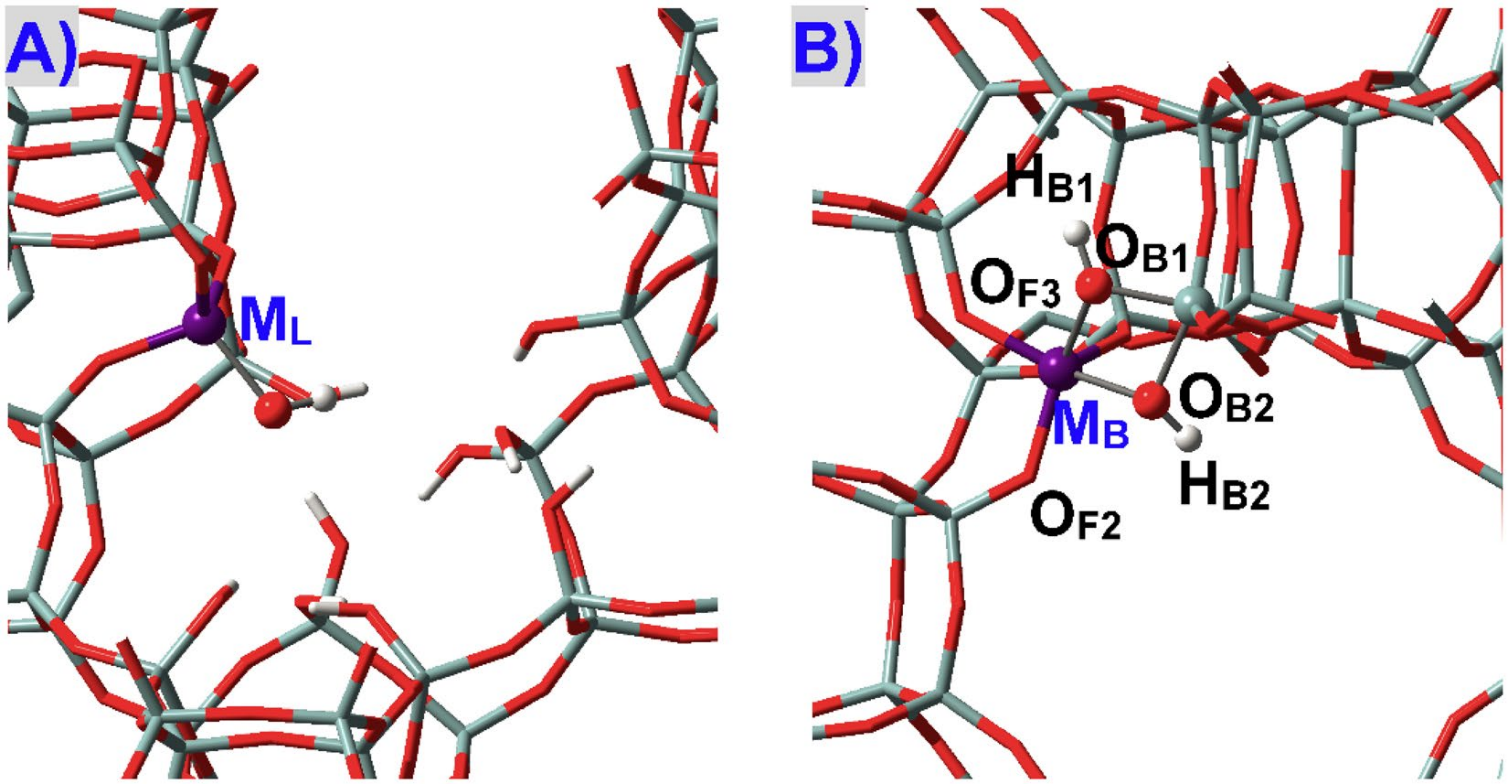
Local structures for defects within M(IV)-BEA zeolites that display (**A**) Lewis acidity (**M**_**L**_) and (**B**) Brϕnsted acidity (**M**_**B**_).

Lewis and Brϕnsted acidic defects in M(IV)-incorporated zeolites were given in Figs 1 and 3. Lewis acidic defects (**M**_**L**_) were constructed by removing a neighboring Si atom as well as its first-shell three Si atoms^26,34,48–50,55^, while Brϕnsted acidic defects (**M**_**B**_) were created with the formation of the ≡M(OH)_2_Si≡ linkage^31,53,58,64^.

### Methods

The Perdew, Burke and Ernzrhof (PBE) exchange-correlation functional^65,66^ supplemented with the damped C_6_ dispersion term^67^ (referred to as DFT-D2, implemented in the VASP software) was used. The standard PAW (projected augmented wave) psuedopotentials were used for all elements, while M(IV) ions are exceptions that were handled by the highest electronic PAW pseudopotentials (Sn_d for Sn, Ti_pv for Ti, Zr_sv for Zr and Ge_d for Ge), in that their semi-core *s*, *p* or *d* states should be regarded as valence electrons^33,37,39,55,68^. The energy cutoff was 400.0 eV, and the Brillouin zone sampling was restricted to Γ-point. Structural optimizations finished when the forces on each atom are below 0.05 eV Å^−1^. The adsorption energies of the N^th^ water within M(IV)-incorporated zeolites were defined as,

1 $${E}_{{\rm{adN}}}=E({\rm{MZeo}}-{{\rm{nH}}}_{2}{\rm{O}})\mbox{--}E({\rm{MZeo}}\,-\,({\rm{n}}-1){{\rm{H}}}_{2}{\rm{O}})-{E}({{\rm{H}}}_{2}{\rm{O}})$$

where MZeo-nH_2_O stands for M(IV)-incorporated zeolites respectively adsorbed with N-numbered water (n = 0, 1, 2, 3, 4). Noting that the *E*_ad2_ calculations were based on the lower-energy MZeo-H_2_O configurations (e.g., **Sn9**_**P**_^**b**^ rather than **Sn9**_**P**_^**a**^ due to the larger *E*_ad1_ value and higher stability, see Table 1).

**Table 1:** M-O_W_ distances (Å) and adsorption energies of the n^th^ water (*E*_adn_, kJ/mol) for defect-free M(IV)-BEA zeolites (M = Sn, Ti, Zr, Ge; n = 1, 2).

		M2(IV)		M9(IV)		M6(IV)	
		M-O_W_	*E*_adn_	M-O_W_	*E*_adn_	M-O_W_	*E*_adn_
Ti	**Ti**_**P**_^a^ (n = 1)	2.356	−44.9	2.320	−48.3	2.494	−36.6
	**Ti**_**P**_^**b**^ (n = 1)			2.314	−51.1	2.343	−48.9
	**Ti**_**P**_^**ab**^ (n = 2)			2.376/3.042	−19.0	3.083/2.394	−23.7
Sn	**Sn**_**P**_^a^ (n = 1)	2.365	−64.4	2.330	−73.4	2.448	−53.9
	**Sn**_**P**_^**b**^ (n = 1)			2.331	−74.2	2.366	−67.1
	**Sn**_**P**_^**ab**^ (n = 2)			2.402/2.354	−42.6	2.363/2.355	−48.8
Ge	**Ge**_**P**_^**a**^ (n = 1)	2.542	−27.9	2.492	−25.2		
	**Ge**_**P**_^**b**^ (n = 1)			2.469	−22.7		
	**Ge**_**P**_^**ab**^ (n = 2)			3.640/2.357	−20.5		
Zr	**Zr**_**P**_^**a**^ (n = 1)	2.425	−64.8	2.388	−78.6		
	**Zr**_**P**_^**b**^ (n = 1)			2.389	−78.8		
	**Zr**_**P**_^**ab**^ (n = 2)			2.419/2.421	−61.0		
Si^**a**^	**Si**_**P**_^**a**^ (n = 1)	3.538	−20.3	3.442	−22.6		
	**Si**_**P**_^**b**^ (n = 1)			3.367	−22.3		

^a^Data for all-siliceous BEA zeolite.

## Results and Discussion

**Figure 4:**
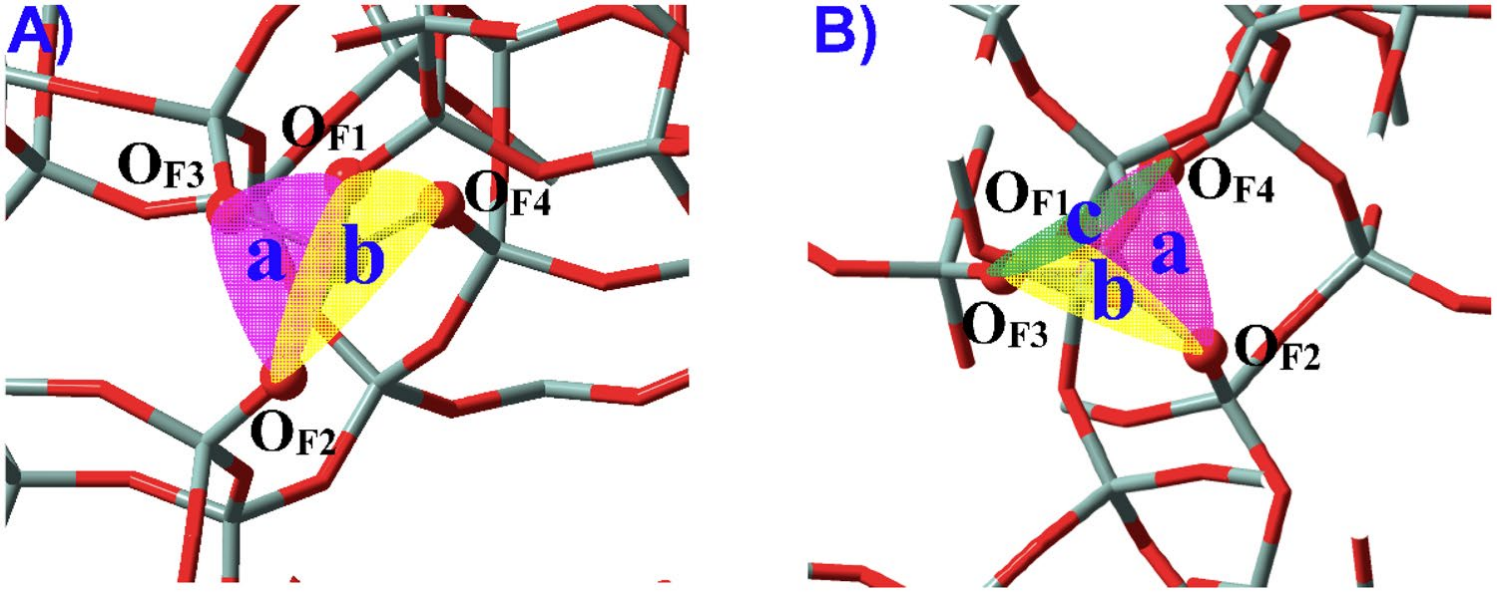
(**A**) Two and (**B**) potential directions present for water molecules to approach the M(IV) sites within zeolites. Different directions are referred to as “a”, “b” and “c” that are colored in semitransparent pink, yellow and green, respectively.

**Figure 5:**
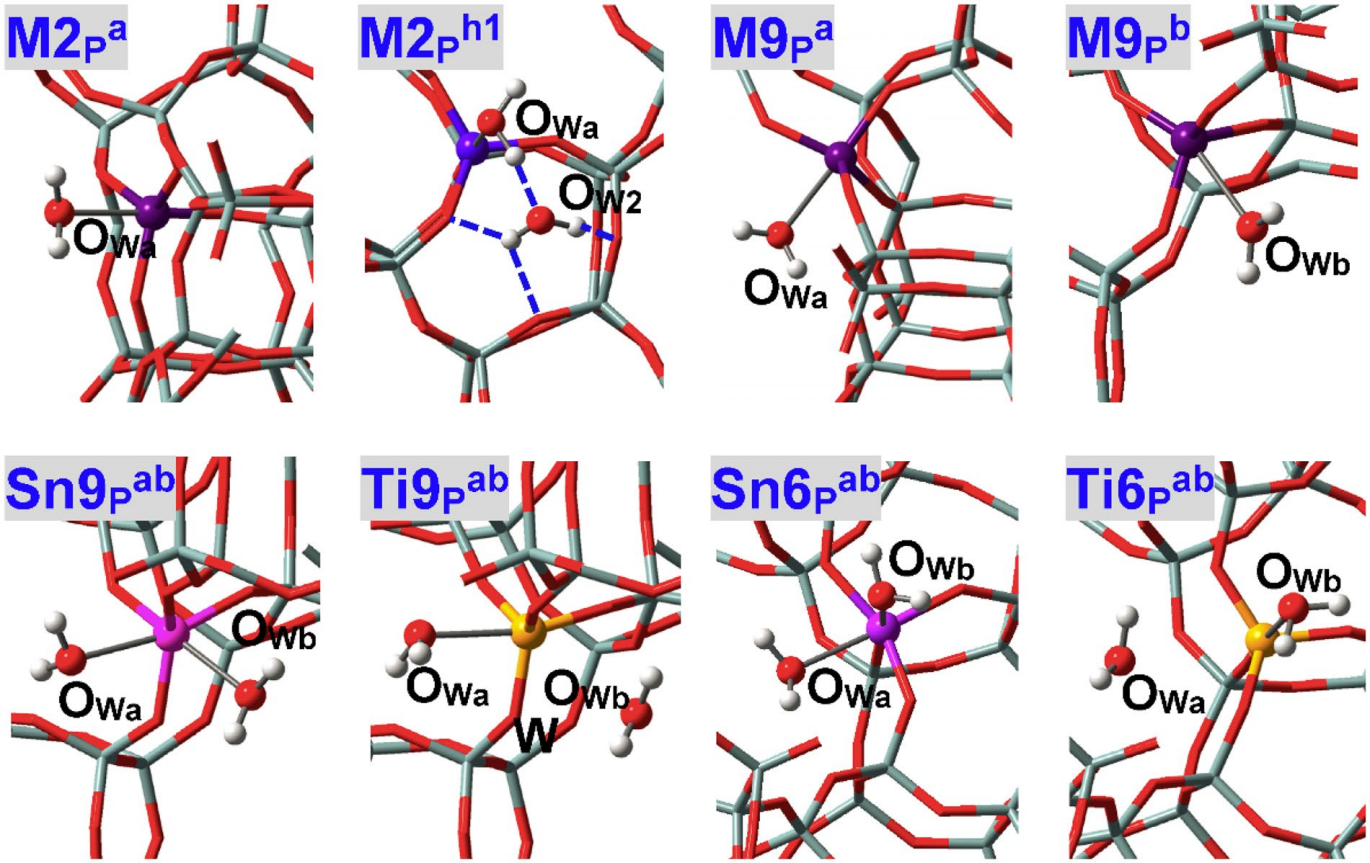
Local configurations of water adsorption at defect-free M2(IV), M9(IV) and M6(IV) sites of BEA zeolites, where M = Sn, Ti, Zr, Ge. O_W2_ in **M2**_**P**_^**h1**^ refers to the water molecule that is stabilized mainly by H-bonds.

As illustrated in Fig. 4, water can approach some T sites of zeolites via different directions that are represented by “a”, “b” and “c”. Nomenclature of adsorption configurations includes such information as M(IV) species (**M**_**P**_, **M**_**L**_, **M**_**B**_, see Fig. 1), number of T sites (1∼9) and direction of water adsorption (a, b, c); e.g., **M9**_**P**_^**ab**^ (Fig. 5) in BEA zeolite stands for the adsorption configuration where two water molecules approach the perfectly tetrahedral M9(IV) site via “a” and “b” directions (M = Sn, Ti, Zr, Ge). Adsorption configurations where water is assumed to form H-bonds with other water molecules and framework-O atoms (referred to as O_F_) are suffixed by hi (i = 1, 2, …), see **M2**_**P**_^**h1**^ in Fig. 5 for instance.

### M(IV)2 Sites in Defect-free BEA Zeolite

The local adsorption configurations of water at defect-free M2(IV)-BEA zeolites are shown in Fig. 5 (**M2**_**P**_^**a**^), and the M-O_Wa_ distances and adsorption energies (*E*_ad1_) are given in Table 1. The M-O_Wa_ distances are 2.356, 2.365, 2.542 and 2.425 Å respectively for M = Ti, Sn, Ge and Zr, in line with previous reports^21–23,25–38^ and indicative of chemisorption. Although with a smaller radius than Sn(IV) (0.53 Å vs. 0.71 Å), Ge(IV) corresponds to larger M-O_Wa_ distances suggesting inferior interactions. The adsorption energies of water (*E*_ad1_) are calculated at −44.9, −64.4, −27.9 and −64.8 kJ/mol respectively for M = Ti, Sn, Ge and Zr, and the interaction strengths within Ge2(IV)-BEA zeolite are almost at the level of all-siliceous zeolite (−20.3 kJ/mol). The adsorption performances of M(IV)-BEA zeolites are in line with their catalytic effects (Zr ≈ Sn > Ti > Ge)^2,3,13–15^, including the results of two-dimensional zeolites available^69^. The thermodynamic changes for the hydrolysis of M-O bonds fall within the range of 63.8∼73.9 kJ/mol, which are obviously lower than that of all-siliceous zeolite (106.2 kJ/mol) and indicate the less stability and more likelihood of forming defects due to M(IV) incorporation.

M(IV)2 sites are situated at the straight channel of BEA zeolite and it is assumed that only one water can be chemisorbed, as testified by adsorption of the second water that forms H-bonds with the first water and O_F_ atoms, see **M2**_**P**_^**h1**^ in Fig. 5. Accordingly, all M(IV)2 active sites are penta-coordinated with adsorption of one water. Strong H-bonds are constructed between two adsorbed water molecules and the O_Wa_H•••O_W2_ distances are 1.684, 1.607, 1.664 and 1.658 Å respectively for M = Ti, Sn, Ge and Zr. The second water is further stabilized by three H-bonds with O_F_ atoms; e.g., the O_W2_H•••O_F_ H-bonds in **Sn2**_**P**_^**h1**^ are 2.005, 2.327 and 2.765 Å. Interestingly, its presence promotes the interaction of M2(IV) sites with the first water, since the M-O_Wa_ distances are respectively 2.255, 2.267, 2.212 and 2.352 Å for M = Ti, Sn, Ge and Zr and all are shorter than those of **M2**_**P**_^**a**^ (Table 1). As a synergy of the two effects, the adsorption energies of the second water in **M2**_**P**_^**h1**^ (*E*_ad2_) are substantial and amount to −58.6, −67.1, −53.0 and −62.1 kJ/mol respectively for M = Ti, Sn, Ge and Zr, which are comparable to, if not larger than, the *E*_ad1_ values of **M2**_**P**_^**a**^ where water is chemisorbed; in addition, the *E*_ad2_ values are close for all M(IV) ions and are not so dependent on the identify of M(IV) ions as those of chemisorbed water (*E*_ad1_).

### M(IV)9 and M(IV)6 Sites in Defect-free BEA Zeolite

M(IV)9 sites in BEA zeolite are at the intersection of two channels and can be approached by water via either “a” or “b” direction, see Figs 4 and 5 (**M9**_**P**_^**a**^ and **M9**_**P**_^**b**^)^33^. For a specific M(IV) ion, the M-O_Wa_ and M-O_Wb_ distances are close to each other suggesting comparable interactions from two directions, as corroborated by the calculated adsorption energies (*E*_ad1_, Table 1). When two water molecules via “a” and “b” directions approach the M9(IV) sites of M9(IV)-BEA zeolites at the same time, both of them construct direct bonds with Sn9(IV) and Zr9(IV) sites while only one remains chemisorbed for Ti9(IV) and Ge9(IV) sites, see Fig. 5 (**Sn9**_**P**_^**ab**^ and **Ti9**_**P**_^**ab**^). That is, the coordination numbers of Sn9(IV), Zr9(IV) and Ti9(IV), Ge9(IV) active sites are expanded respectively to six and five, in line with the results of dynamic nuclear polarization surface enhanced NMR spectra of Sn-BEA zeolite and (resonant) valence-to-core X-ray emission spectra of TS-1 zeolite^61,62^. The adsorption energies of the second water (*E*_ad2_) in **M9**_**P**_^**ab**^ are calculated to be −19.0, −42.6, −20.5 and −61.0 kJ/mol respectively for M = Ti, Sn, Ge and Zr, consistent with the changing trends of M-O_W_ distances (Table 1). Owing to competition of two chemisorbed water, the *E*_ad2_ values in **Sn9**_**P**_^**ab**^ and Zr**9**_**P**_^**ab**^ reduce to some extent as compared to the *E*_ad1_ values, while the *E*_ad2_ value of **Ti9**_**P**_^**ab**^ due to mainly H-bonding interactions descends substantially and is close to those of all-siliceous zeolite (Table 1).

M(IV)6 sites at the intersection of BEA zeolite are also investigated that substantialize the results of M9(IV) sites: Sn6(IV) and Ti6(IV) sites can chemisorb at most two and one water molecules, respectively, see their local structures in Fig. 5 (**Sn6**_**P**_^**ab**^ and **Ti6**_**P**_^**ab**^) and geometric and energetic data in Table 1. That is, Sn6(IV) active sites adopt the octahedral geometry with adsorption of two water molecules while Ti6(IV) active sites are penta-coordinated with adsorption of one water.

### Adsorption with More Water Molecules

**Figure 6:**
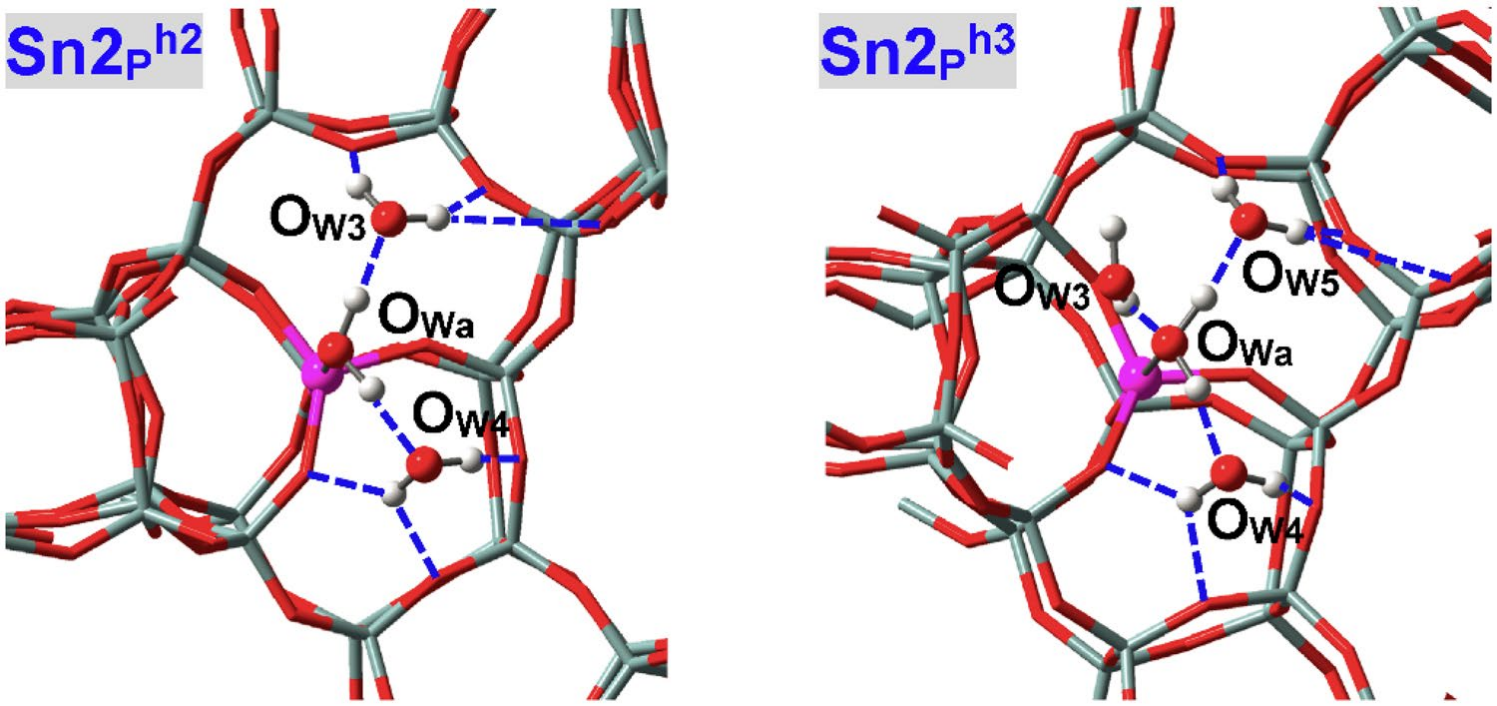
Local adsorption configurations for three and four water molecules within defect-free Sn2(IV)-BEA zeolite. O_W3_ and O_W4_ refer to the water molecules that are stabilized mainly by H-bonds.

Figure 6 depicts the local configurations of Sn2(IV)-BEA zeolite adsorbed with three (**Sn2**_**P**_^**h2**^) and four (**Sn2**_**P**_^**h3**^) water molecules. In **Sn2**_**P**_^**h2**^, the second and third water molecules are located at either side of the chemisorbed one and their chemical environments resemble each other. The O_Wa_H•••O_W3_ and three O_W3_H•••O_F_ H-bonds are 1.603 Å and 2.134, 2.163, 2.961 Å, respectively. The Sn2-Ow_a_ distance in **Sn2**_**P**_^**h2**^ equals 2.186 Å and is further shortened than in **Sn2**_**P**_^**h1**^ (2.267 Å); nonetheless, *E*_ad3_ (−64.2 kJ/mol) decreases slightly as compared to *E*_ad2_, due to the inferior H-bonding interactions as reflected by their distances. The *E*_ad4_ value (−33.5 kJ/mol) of **Sn2**_**P**_^**h3**^ shows considerable reductions albeit the fourth water also constructs H-bonds with the chemisorbed water, due to the obviously weaker H-bonding interactions; e.g., the O_W4_H•••O_Wa_ distance equals 1.971 Å and is apparently longer than those of the second and third water molecules. Subsequent adsorption constructs the second, third and higher-order water shells around the chemisorbed one, which will not be discussed here.

**Figure 7:**
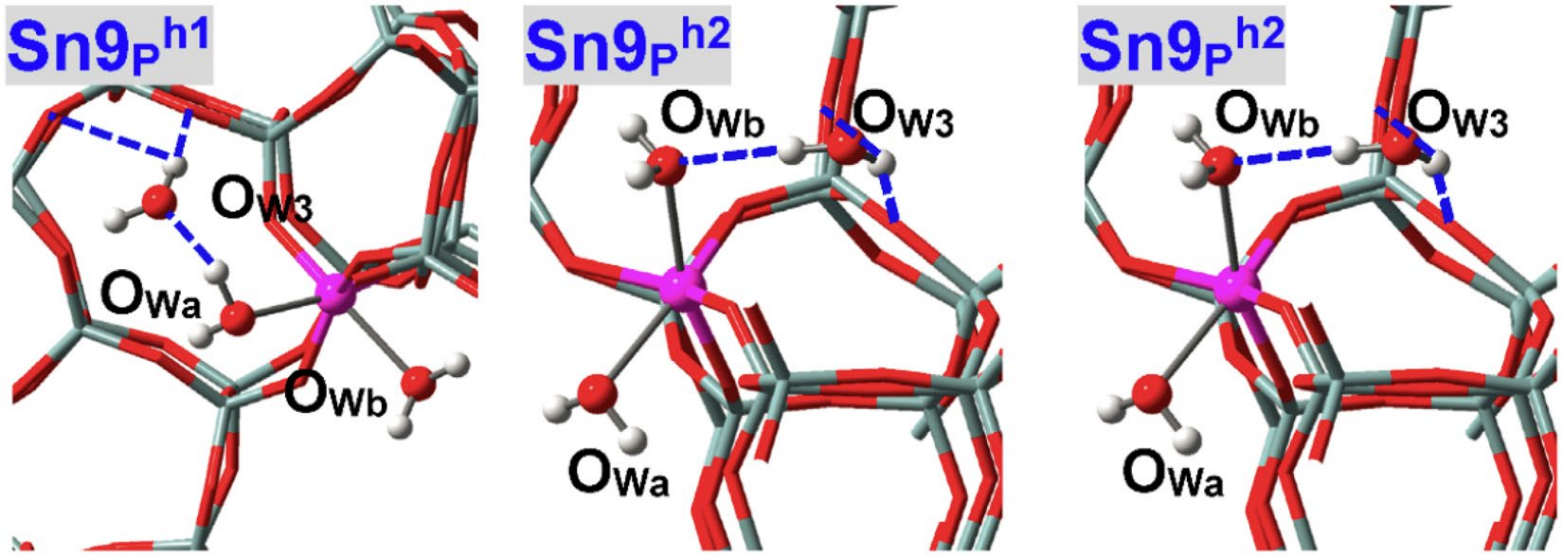
Local adsorption configurations for three water molecules within defect-free Sn9(IV)-BEA zeolite. O_Wi_ in **Sn9**_**P**_^**abi**^ refers to the water molecule in-between O_Wa_ and O_Wb_, and O_W3_ in **Sn9**_**P**_^**h1**^ and **Sn9**_**P**_^**h2**^ stands for the water molecule that is stabilized mainly by H-bonds.

H-bonded adsorption configurations of Sn9(IV) site are shown in Fig. 7 (**Sn9**_**P**_^**h1**^ and **Sn9**_**P**_^**h2**^) that resemble the condition of Sn2(IV) site. The third water in **Sn9** ^**h1**^ rather than in **Sn9** ^**h2**^ is more stabilized by H-bonds, and the O_Wa_H•••O_W3_ and O_Wb_H•••O_W3_ distances are optimized at 1.677 and 2.151 Å, respectively. The third water in **Sn9** ^**h1**^ facilitates the chemisorption of associated water (Sn9-O_Wa_: 2.289 Å in **Sn9** ^**h1**^ vs. 2.402 Å in **Sn9**_**P**_^**ab**^) while in **Sn9**_**P**_^**h2**^ plays an adverse effect for the chemisorption of associated water (Sn9-O_Wb_: 2.425 Å in **Sn9**_**P**_^**h2**^ vs. 2.354 Å in **Sn9**_**P**_^**ab**^). As a result, the adsorption energies of the third water (*E*_ad3_) differ markedly for **Sn9**_**P**_^**h1**^ and **Sn9**_**P**_^**h2**^ that amount to −61.2 and −26.9 kJ/mol, respectively. It is interesting to find that three water molecules can also be simultaneously chemisorbed at Sn9(IV) site with elongated Sn-O_W_ distances (2.665, 2.558 and 2.409 Å)^70^, see **Sn9**_**P**_^**abi**^ in Fig. 7 and Table 2. However, the *E*_ad3_ value of **Sn9**_**P**_^**abi**^ equals −26.6 kJ/mol and is apparently less than that of **Sn9** ^**h1**^. That is, the coordination number of Sn9(IV) site in BEA zeolite should predominate as six although potentially expanded to seven. Parallel p-DFT calculations are conducted for Zr9(IV)-BEA zeolite and it indicates that its coordination number can also be expanded to seven with the Zr-O_w_ distances of 2.518, 2.518 and 2.454 Å (Table 2). The *E*_ad3_ difference of **Zr9**_**P**_^**abi**^ vs. **Zr9**_**P**_^**h1**^ (−36.5 vs. −59.8 kJ/mol) is less than that of **Sn9**_**P**_^**abi**^ vs. Sn**9**_**P**_^**h1**^, suggesting that Zr9(IV)-BEA zeolite has a greater possibility of developing the hepta-coordination mode.

**Table 2:** M-O_W_ distances (Å), adsorption energies of the 3^th^ water (*E*_adn_, kJ/mol) for M9(IV)-BEA zeolites (M = Sn, Zr).

	**M9** ^**abi**^ (n = 3)		**M9** ^**h1**^ (n = 3)	
	M-O_Wa_/M-O_Wb_/M-O_Wc_^a^	*E*_ad3_	M-O_Wa_/M-O_Wb_/M-O_W3_^a^	*E*_ad3_
**Sn**_**P**_	2.665/2.558/2.409 (2.544)	−26.6	2.289/2.361/4.095 (2.325)	−61.2
**Zr**_**P**_	2.518/2.518/2.454 (2.497)	−36.5	2.357/2.409/4.267 (2.383)	−59.8
**Sn**_**L**_	2.445/2.446/2.568 (2.486)	−16.6	2.221/2.346/3.577 (2.283)	−66

^a^Average M-O_W_ distances corresponding to chemisorbed water are given in parentheses.

### Defect-free CHA and FER Zeolites

**Figure 8:**
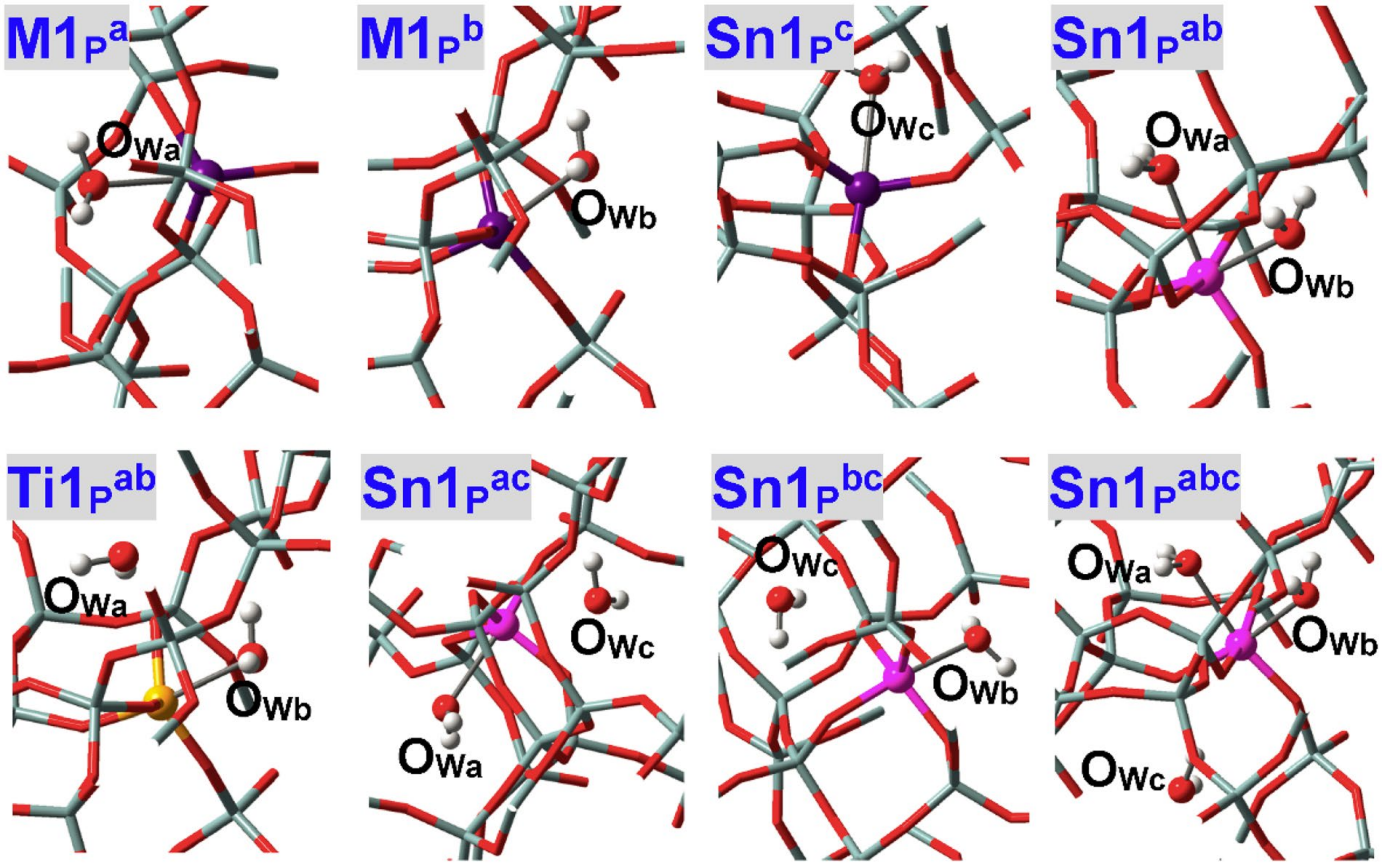
Local configurations for water adsorption within defect-free M(IV)-CHA zeolites, where M = Sn, Ti.

Figure 8 and Table 3 indicate that water can approach M(IV) sites of CHA zeolite from three different directions (“a”, “b” and “c” as shown in Fig. 4). Each direction in Sn(IV)-CHA zeolite results in the formation of direct Sn-O_W_ bonds (Sn-O_Wa_: 2.492 Å; Sn-O_Wb_: 2.395 Å; Sn-O_Wc_: 2.443 Å) while only two directions in Ti(IV)-CHA zeolite can be chemisorbed with water (Ti-O_Wa_: 2.905 Å; Ti-O_Wb_: 2.371 Å; Ti-O_Wc_: 2.414 Å)^21–23,25–28,31–33^, where the Ti-O_Wa_ distance is apparently longer. The adsorption energies of three directions (*E*_ad1_) differ remarkably and are maximal for **M1**_**P**_^**b**^ with the values being −72.3 and −57.1 kJ/mol for M = Sn and Ti, respectively (Table 3). For adsorption of two water molecules in Sn(IV)-CHA zeolite, **Sn1**_**P**_^**ab**^ remains hexa-coordinated (Sn-O_w_: 2.457 and 2.261 Å) while **Sn1**_**P**_^**ac**^ (Sn-O_w_: 2.565 and 3.033 Å) and **Sn1**_**P**_^**bc**^ (Sn-O_w_: 2.307 and 3.524 Å) transform to penta-coordination. Instead, all adsorption configurations of Ti(IV)-CHA zeolite are presented as penta-coordination including the one corresponding to **Sn1**_**P**_^**ab**^ (i.e., **Ti1**_**P**_^**ab**^ with Ti-O_w_ distances of 3.699 and 2.347 Å). Chemisorption of three water molecules in Sn(IV)-CHA zeolite seems impossible, see the optimized structure (**Sn1**_**P**_^**abc**^) in Fig. 8. In consequence, Sn(IV) and Ti(IV) active sites of CHA zeolites are respectively hexa- and penta-coordinated that resemble the condition of BEA zeolites.

**Table 3:** M-O_W_ distances (Å), adsorption energies of the n^th^ water (*E*_adn_, kJ/mol) for defect-free M(IV)-CHA and M(IV)-FER zeolites (M = Sn, Ti; n = 1, 2).

	**M1**_**P**_^**a**^ (n = 1)		**M1**_**P**_^**b**^ (n = 1)		**M1**_**P**_^**c**^ (n = 1)		**M1**_**P**_^**ab**^ (n = 2)	
	M-O_Wa_	*E*_ad1_	M-O_Wb_	*E*_ad1_	M-O_Wc_	*E*_ad1_	M-O_Wa_/M-O_Wb_	*E*_ad2_
Sn-CHA	2.492	−39.4	2.395	−72.3	2.443	−42.4	2.457/2.261	−46.8
Ti-CHA	2.905	−24.9	2.371	−57.1	2.414	−47.5	3.699/2.347	−37.6
Sn-FER	2.398	−720	2.398	−690			2.379/2.392	−65.9
Ti-FER	2.392	−54.7	2.449	−40.6			2.365/2.419	−41.4

**Figure 9:**
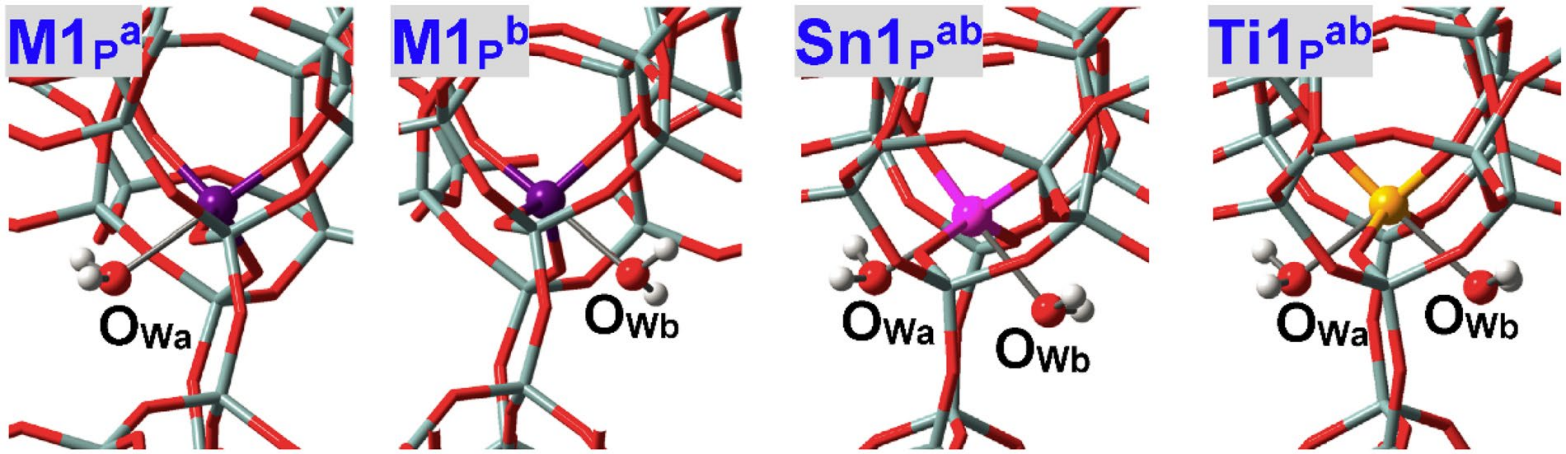
Local configurations for water adsorption within defect-free M(IV)-FER zeolites, where M = Sn, Ti.

Figure 9 shows the adsorption configurations of water within M(IV)-FER zeolites. Water can be chemisorbed at the M(IV) sites of FER zeolite from two different directions referred to as “a” and “b”, and the Sn-O_wa_, Ti-O_wa_, Sn-O_wb_ and Ti-O_wb_ distances in **Sn1**_**P**_^**a**^, **Ti1**_**P**_^**a**^, **Sn1**_**P**_^**b**^ and **Ti1**_**P**_^**b**^ are respectively 2.398, 2.392, 2.398 and 2.449 Å. Chemisorption of two water molecules is viable in Sn(IV)-FER zeolite as in Sn(IV)-BEA and Sn(IV)-CHA zeolites (Sn-O_w_: 2.379 and 2.392 Å), implying that chemisorption of the first water facilitates the interaction of the second water as verified subsequently. It is surprising to find that two water molecules can be chemisorbed at the Ti(IV) sites of FER zeolite, and the Ti-O_wa_ and Ti-O_wb_ distances in **Ti1**_**P**_^**ab**^ are 2.365 and 2.419 Å that are also shorter than those adsorbed with one water (Table 3). Accordingly, both Sn(IV) and Ti(IV) active sites in FER zeolites adopt the octahedral geometry with adsorption of two water molecules. As indicated in Table 3, the adsorption energies of the second water (*E*_ad2_) are comparable to those of the first water (*E*_ad1_) from the same direction (e.g., “b” direction for **Ti1**_**P**_^**ab**^ and **Ti1**_**P**_^**b**^).

Figure 4A shows that “a” and “b” directions each have three different O_F_O_F_O_F_M dihedrals, and the accessibility of water to M(IV) sites is estimated by the minimal dihedral^33^,

2 $${{\rm{\Omega }}}_{{\rm{\min }}}=\,{\rm{\min }}\,[{\rm{\Omega }}({{\rm{O}}}_{{\rm{F}}1}{{\rm{O}}}_{{\rm{F}}2}{{\rm{O}}}_{{\rm{F}}3}{\rm{M}}),{\rm{\Omega }}({{\rm{O}}}_{{\rm{F}}2}{{\rm{O}}}_{{\rm{F}}3}{{\rm{O}}}_{{\rm{F}}1}{\rm{M}}),{\rm{\Omega }}({{\rm{O}}}_{F3}{{\rm{O}}}_{{\rm{F}}1}{{\rm{O}}}_{{\rm{F}}2}{\rm{M}})]$$

3 $${{\rm{\Psi }}}_{{\rm{\min }}}=\,{\rm{\min }}\,[{\rm{\Psi }}({{\rm{O}}}_{{\rm{F}}1}{{\rm{O}}}_{{\rm{F}}4}{{\rm{O}}}_{{\rm{F}}2}{\rm{M}}),{\rm{\Psi }}({{\rm{O}}}_{{\rm{F}}4}{{\rm{O}}}_{{\rm{F}}2}{{\rm{O}}}_{{\rm{F}}1}{\rm{M}}),{\rm{\Psi }}({{\rm{O}}}_{{\rm{F}}2}{{\rm{O}}}_{{\rm{F}}1}{{\rm{O}}}_{{\rm{F}}4}{\rm{M}})]$$

Chemisorption of one water transforms the M(IV) geometry from tetrahedral to bipiramidal^21,23,33^, and the dihedral of direction with water adsorption reduces substantially while that of the other direction ascends considerably. Chemisorption of the second water causes the Ω_min_ and Ψ_min_ values to again get close to each other and reduce pronouncedly due to the formation of octahedral geometry^61^, see the Ω_min_ and Ψ_min_ values for Sn9(IV) sites of BEA zeolite in Table 4. In M(IV)-FER zeolites, however, both Ω_min_ and Ψ_min_ decline due to adsorption of the first water although to an apparently less extent for the other direction with no water (Table 4), and the smaller dihedral of the other direction indicates that adsorption of the first water facilitates the accessibility of the second water towards M(IV) sites, which is distinct from the condition of other zeolites. It thus deciphers why two water molecules can be chemisorbed at Ti(IV) sites of FER rather than other zeolites.

**Table 4:** The O_F_O_F_O_F_M dihedrals corresponding to the “a” (Ω_min_, degrees) and “b” (Ω_min_, degrees) directions for defect-free M(IV)-incorporated zeolites adsorbed with water molecules (M = Sn, Ti; n = 0, 1, 2)^a^.

	**M**_**P**_ (n = 0)		**M**_**P**_^a^ (n = 1)		**M**_**P**_^**b**^ (n = 1)		**M**_**P**_^**ab**^ (n = 2)	
	Ω_min_	Ψ_min_	Ω_min_	Ψ_min_	Ω_min_	Ψ_min_	Ω_min_	Ψ_min_
Sn9-BEA	33.43	33.13	15.33	41.07	40.52	15.88	21.04	17.71
Ti9-BEA	33.43	33.13	17.23	39.31	38.68	16.97	20.29	32.54 ^*b*^
Sn-CHA	33.71	32.27	19.17	34.96	36.98	16.94	13.27	10.74
Ti-CHA	33.71	32.27	29.15	30.25	39.01	16.06	37.48 ^*b*^	15.56
Sn-FER	35.86	36.50	19.77	34.35	33.97	20.04	16.83	17.77
Ti-FER	35.86	36.50	19.67	35.46	34.51	22.38	18.16	20.17

^a^The number of adsorbed water molecules is referred to as “n”; ^b^No direction interaction between the Ti(IV) site and adsorbed water molecule.

### Lewis Acidic Defects

**Figure 10:**
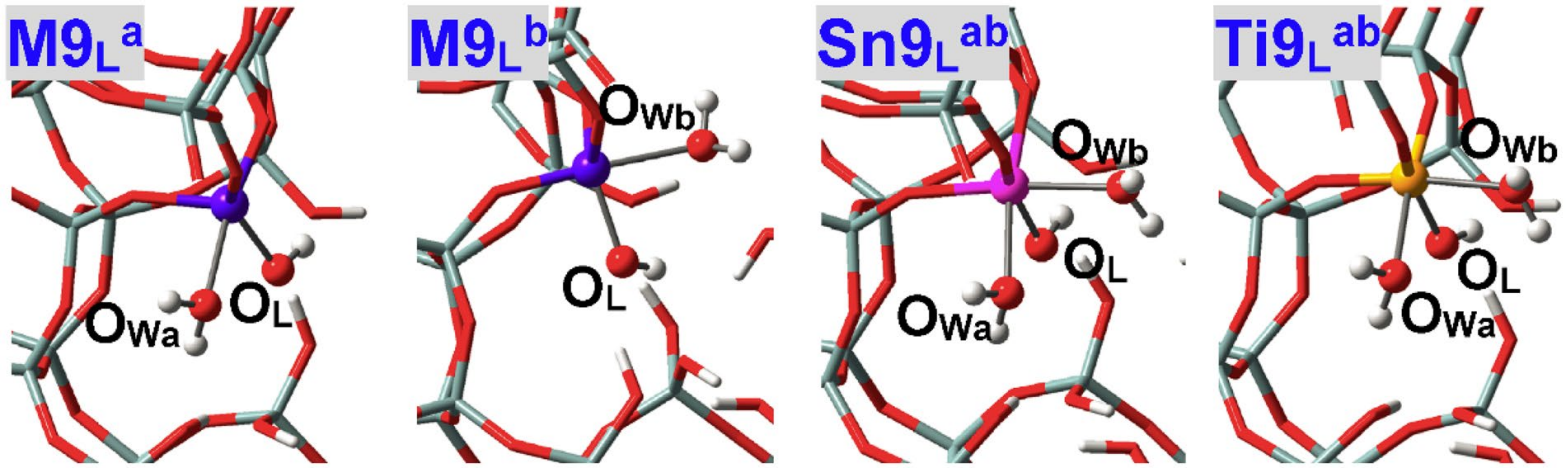
Local adsorption configurations for one and two water molecules at M9(IV) Lewis acidic defects (**M9**_**L**_) of BEA zeolites, where M = Sn, Ti.

**Figure 11:**
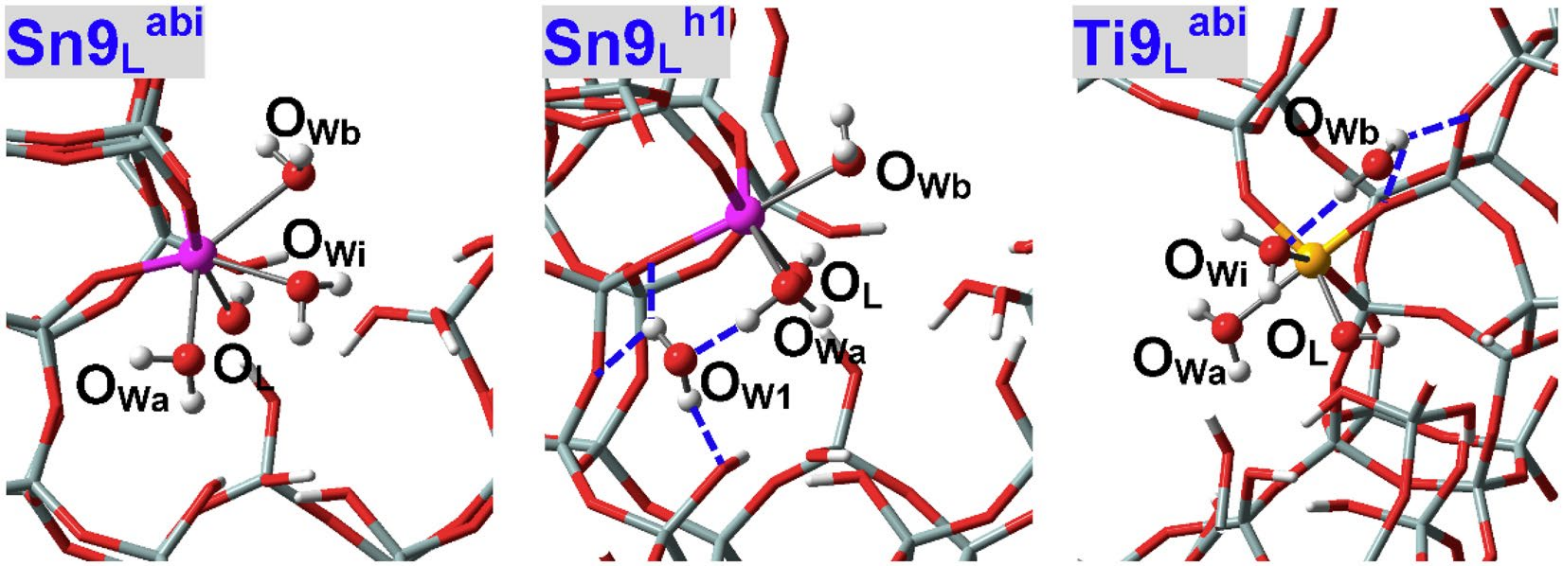
Local adsorption configurations for three water molecules M9(IV) Lewis acidic defects (**M9**_**L**_) of BEA zeolites, where M = Sn, Ti. O_Wi_ in **Sn9**_**L**_^**abi**^ refers to the water molecule in-between O_Wa_ and O_Wb_, and O_W3_ in **Sn9**_**L**_^**h1**^ and **Sn9**_**L**_^**h2**^ stands for the water molecule that is stabilized mainly by H-bonds. In **Ti9**_**L**_^**abi**^, three water molecules are assumed to form direct bonds as in **Sn9**_**L**_^**abi**^.

Defects have been implicated to be critical for catalytic reactions^54–60^, and is this effect associated, at least in part, with alteration of the active sites? The adsorption configurations of water at M9(IV) Lewis acidic defects (**M9**_**L**_) of BEA zeolites are shown in Fig. 10. Water can approach M9(IV) Lewis acidic defects from “a” and “b” directions as in the condition of perfectly tetrahedral sites (**M9**_**P**_) while interactions are reinforced (Table 5); e.g., the adsorption energies (*E*_ad1_) are −85.1 and −89.1 kJ/mol respectively for **Sn9**_**L**_^**a**^ and **Sn9**_**L**_^**b**^ and surpass those of **Sn9**_**P**_^**a**^ and **Sn9**_**P**_^**b**^ (Table 1)^49,55^. More significant promotion effects of Lewis acidic defects are detected during adsorption of the second water: The *E*_ad2_ value in **Sn9**_**L**_^**ab**^ equals −78.6 kJ/mol and is even larger than the *E*_ad1_ values of perfectly tetrahedral sites. Two water molecules can be chemisorbed at Ti9(IV) Lewis acidic defects (Ti-O_W_ distances of **Ti9**_**L**_^**ab**^: 2.278 and 2.233 Å), and the *E*_ad2_ value (−55.0 kJ/mol) surpasses the *E*_ad1_ values of perfectly tetrahedral Ti9(IV) sites (Table 1), which agree with the results of Sn9(IV) Lewis acidic defects. The adsorption configurations of three water molecules at Sn9(IV) Lewis acidic defects (Fig. 11) are close to those of perfectly tetrahedral sites, and the *E*_ad3_ values are calculated at −16.6 and −66.0 kJ/mol for **Sn9**_**L**_^**abi**^ and **Sn9**_**L**_^**h1**^, respectively (Table 2). Accordingly, Lewis acidic defects inhibit somewhat the formation of hepta-coordinated Sn9(IV) species (Chemisorption of three water molecules is also tried for Ti9(IV) Lewis acidic defects, while only two water molecules remain bonded and the third water forms H-bonds with other water molecules and O_F_ atoms, see Fig. 11 (**Ti9Labi**)).

**Table 5:** M-O_W_ distances (Å) and adsorption energies of the n^th^ water (*E*_adn_, kJ/mol) for M9(IV) Lewis acidic defects of BEA zeolites (M = Sn, Ti; n = 1, 2).

	**M9**_**L**_^a^ (n = 1)		**M9**_**L**_^b^ (n = 1)		**M9**_L_^ab^ (n = 2)	
	M-O_W_	*E*_ad1_	M-O_W_	*E*_ad1_	M-O_W_	*E*_ad2_
**Sn**_**L**_	2.323	−85.1	2.341	−89.1	2.325/2.298	−78.6
**Ti**_**L**_	2.288	−56.7	2.289	−61.7	2.278/2.233	−55

Table 6 lists the root-mean-square deviations (RMSD) of local M(IV) sites during water adsorption. For each M(IV) site, RMSDs generally increase with the number of chemisorbed water molecules, implying larger structural perturbations; in addition, at a specific chemisorbed water content, Lewis acidic defects always result in obviously higher RMSDs than corresponding perfectly tetrahedral sites; e.g., the RMSDs are calculated to be 0.20 and 0.24 Å in **Sn9**_**P**_^**a**^ and **Sn9**_**P**_^**ab**^ while are enlarged to 0.34 and 0.47 Å in **Sn9**_**L**_^**a**^ and **Sn9**_**L**_^**ab**^, respectively. Accordingly, the more structural flexibility of Lewis acidic defects facilitates the interaction with water and allows the formation of hexa-coordinated Ti(IV) species in BEA zeolite.

**Table 6:** Root-mean-square deviations (RMSD, Å) for M9(IV) active sites of BEA zeolites due to water adsorption (M = Sn, Ti)^a,b^.

	M9^a^	M9^b^	M9^ab^	M9^abi^	M9^h1^
**Sn9**_**P**_	0.2	0.18	0.24	0.34	0.28
**Sn9**_**L**_	0.34	0.31	0.47	0.46	0.52
**Ti9**_**P**_	0.16	0.16	0.15 ^*c*^		
**Ti9**_**L**_	0.29	0.33	0.47		

^a^M(OSi)_4_ and (SiO)_3_MOH are used for calculating the RMSDs of perfect tetrahedral M(IV) sites (**M**_P_), and defects with Lewis acidity (**M**_**L**_), respectively; ^b^The dehydrated M(IV) sites (n = 0) are used as benchmarks; ^c^The second water is not directly associated with the **Ti9**_**P**_ site.

### Brϕnsted Acidic Defects

**Figure 12:**
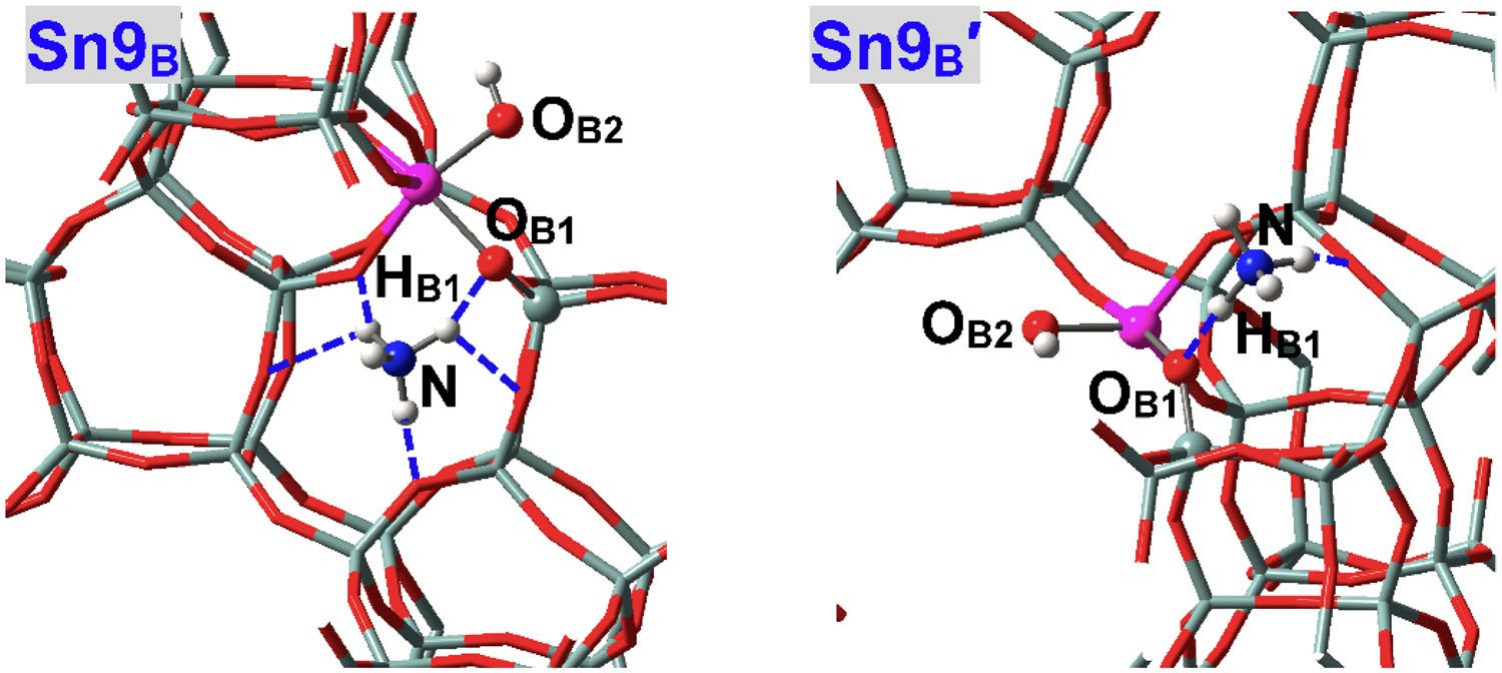
Local configurations for NH_3_ adsorption at two types of Sn9(IV) Brϕnsted acidic defects (**Sn9**_B_ and **Sn9**_B_ **′**) and of Sn-BEA zeolite.

**Figure 13:**
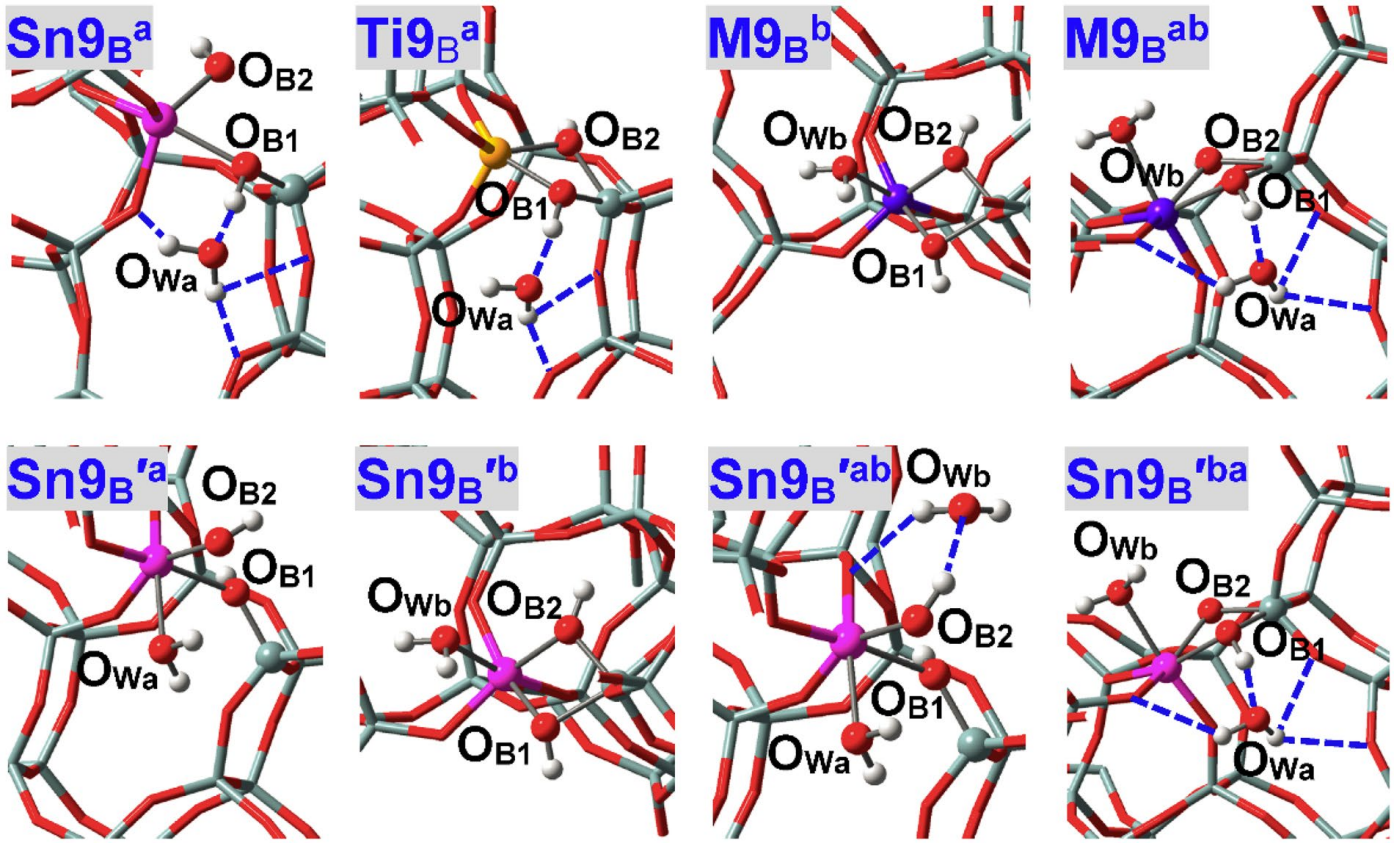
Local configurations for water adsorption at M9(IV) Brϕnsted acidic defects (**M9**_**B**_) of BEA zeolites, where M = Sn, Ti. There emerges a second Sn(IV) Brϕnsted acidic defect named **Sn9**_**B**_ **′**, where O_B2_ forms direct bonds only with the Sn(IV) sites. Both **Sn9**_**B**_ **′** ^**ab**^ and **Sn9**_**B**_ **′** ^**ba**^ are adsorption configurations with two water molecules while the chemisorbed water are from the “a” and “b” directions, respectively.

The adsorption configurations of NH_3_ at defect **M9**_**B**_ of BEA zeolite with the ≡M(OH)_2_Si≡ linkage (Figs 1 and 3) are given in Fig. 12. Defect **M9**_**B**_ in BEA zeolite transfers the proton to NH_3_ automatically forming NH_4_^+^ and thus shows Brϕnsted acidity, which are in line with the results of M(IV)-incorporated MFI zeolites^32,64^. The bridging hydroxyls of defect **M**_**B**_ significantly accelerate the isomerization reaction of glucose to fructose^58^. Figure 13 depicts the adsorption configurations of water at M9(IV) Brϕnsted acidic defects of BEA zeolites, with structural parameters and adsorption energies (*E*_adn_) being listed in Table 7. The M-O_B_ and Si-O_B_ distances are altered but not markedly during water adsorption. The Si-O_B_ distances are obviously elongated than in perfectly tetrahedral Si(IV) sites (ca. 1.62 Å) while M(IV) sites remain tightly bonded with two bridging O_B_ atoms. **Sn9**_**B**_^**a**^ is an exception where the Si-O_B2_ bond is ruptured and the Sn-O_B_ distances reduce considerably. The M-O_W_ distances in **M9**_**B**_^**b**^ are shorter than in perfectly tetrahedral M(IV) sites and hence interactions with water are enhanced as verified by adsorption energies (e.g., *E*_ad1_ = −91.3 kJ/mol for **Sn9**_**B**_^**b**^ vs. −74.2 kJ/mol for **Sn9**_**P**_^**b**^). Formation of Brϕnsted acidic defects (**M**_**B**_) facilitates the accessibility of water to M(IV) sites as reflected by the significantly smaller dihedrals than in perfectly tetrahedral sites: The O_F3_O_B1_O_B2_M dihedrals (Fig. 3) are 10.06° and 10.78° respectively for **Sn9**_**B**_ and **Ti9**_**B**_. However, limited space is available for the second water and as a result, water from “a” direction forms only H-bonds instead of being chemisorbed: The O_B1_H_B1_•••O_Wa_ and O_Wa_H•••O_F_ H-bonds are respectively 1.499 and 1.918, 2.836, 2.670 Å in **Sn9**_**B**_^**a**^ while 1.662 and 2.139, 2.573 Å in **Ti9**_**B**_^**a**^, and stronger O_B1_H_B1_•••O_Wa_ H-bond in **Sn9**_**B**_^**a**^ results from higher Brϕnsted acidity^32^ that agrees with larger O_B1_-H_B1_ distances (1.053 vs. 1.011 Å). The *E*_ad1_ values are −101.3 and −69.2 kJ/mol respectively for **Sn9**_**B**_^**a**^ and **Ti9**_**B**_^**a**^, and structural distortions in **Sn9**_**B**_^**a**^ may facilitate the interactions as testified subsequently. For both **Sn9**_**B**_^**ab**^ and **Ti9**_**B**_^**ab**^, one water is chemisorbed while the other is H-bonded.

**Table 7:** M-O_W_, M-O_B_ and Si-O_B_ distances (Å) and adsorption energies of the n^th^ water (*E*_adn_, kJ/mol) for M9(IV) Brϕnsted acidic defects (**M9**_B_) of BEA zeolites (M = Sn, Ti; n = 1, 2)^a^.

	M-O_W_	M-O_B1_	M-O_B2_	Si-O_B1_	Si-O_B2_	*E*_adn_
**Sn9**_**B**_ (n = 0)		2.106 (2.277)	2.116 (1.975)	1.810 (1.681)	1.861 (3.136)	
**Sn9**_**B**_^**a**^ (n = 1)	3.763 (2.304)	2.210 (2.285)	1.977 (1.998)	1.657 (1.662)	3.121 (3.804)	−101.3 (−8.5)^b^
**Sn9**_**B**_^**b**^ (n = 1)	2.298 (2.298)	2.119 (2.119)	2.123 (2.123)	1.770 (1.770)	1.885 (1.885)	−91.3 (−68.5)
**Sn9**_**B**_^**ab**^ (n = 2) ^c^	2.287/3.554 (2.299/3.598) [2.287/3.554]	2.119 (2.288) [2.119]	2.095 (1.979) [2.095]	1.791 (1.659) [1.791]	1.840 (3.804) [1.840]	−53.9 (−48.5) ^d^ [−63.9]^d^
**Ti9**_**B**_ (n = 0)		2.047	2.030	1.759	1.847	
**Ti9**_**B**_^**a**^ (n = 1)	3.843	2.008	2.039	1.797	1.785	−58.0
**Ti9**_**B**_^**b**^ (n = 1)	2.295	2.064	2.048	1.735	1.860	−69.2
**Ti9**_**B**_^**ab**^ (n = 2)	2.275/3.668	2.062	2.021	1.744	1.824	−56.9

^a^Data for **Sn9**_B_′ (The second form of Brϕnsted acidic defects where O_B2_ form direct bonds only with the Sn(IV) site) are given in parentheses.

^b^The adsorption energy based on **Sn9**_B_^**a**^ is calculated to be −78.5 kJ/mol.

^c^Data for **Sn9**_B_′^**ba**^ are given in brackets.

^d^The adsorption energies of the second water (*E*_ad2_) in **Sn9**_B_′^**ab**^ and **Sn9**_B_ **′** ^**ba**^ are calculated on basis of **Sn9**_B_′^**a**^ and **Sn9**_B_′^**b**^, respectively.

The Si-O_B2_ bond in **Sn9**_**B**_^**a**^ is broken, and such defect referred to as **Sn9**_**B**_ **′** also exists in Sn(IV)-BEA zeolite. In addition, **Sn9**_**B**_ **′** is more stable than **Sn9**_**B**_ and their energy difference equals −22.8 kJ/mol; however, **Ti9**_**B**_ **′** is non-existent and spontaneously transforms to **Ti9**_**B**_. The O_B2_H_B2_ group in **Sn9**_**B**_ **′** is flexible and allows water chemisorption from either “a” or “b” direction, see **Sn9**_**B**_ **′** ^**a**^ and **Sn9**_**B**_ **′** ^**b**^ in Fig. 13. Chemisorption of water from “b” direction causes structural reconstruction to resemble **Sn9**_**B**_^**b**^. The adsorption energies (*E*_ad1_) in **Sn9**_**B**_ **′** ^**a**^ and **Sn9**_**B**_ **′** ^**b**^ are respectively −8.5 and −68.5 kJ/mol, and the particularly small value in **Sn9**_**B**_ **′** ^**a**^ is caused by the serious structural distortion in order to accommodate the chemisorbed water. Accordingly, water from “a” direction of **Sn9**_**B**_ **′** should be preferentially the H-bonded adsorption configuration (i.e., **Sn9**_**B**_^**a**^), and the *E*_ad1_ value calculated this way amounts to −78.5 kJ/mol (Table 7). Similar to the condition of **Sn9**_**B**_, only one water can be chemisorbed at **Sn9**_**B**_ **′**, see Fig. 13 (**Sn9**_**B**_ **′** ^**ab**^ and **Sn9**_**B**_ **′** ^**ba**^ where water is chemisorbed from “a” “b” directions, respectively). Accordingly, M(IV) Brϕnsted acidic defects, for both acidic forms and different M(IV) ions, result in only the hexa-coordination mode with chemisorption of one water.

## Conclusions

Periodic density functional theory (p-DFT) calculations have been used to comprehensively investigate the active sites of M(IV)-incorporated zeolites, considering the identity of M(IV) ions, topology of zeolites, type of framework species and choice of T sites.

With regard to defect-free BEA zeolites, all M(IV) active sites are penta-coordinated with chemisorption of one water when situated at the straight channel while divergences arise when situated at the intersection: Sn(IV) and Zr(IV) active sites predominate as hexa-coordination while Ti(IV) and Ge(IV) active sites remain penta-coordinated; in addition, it is surprising to find that Sn(IV) and Zr(IV) are potentially expanded to hepta-coordination although with relatively small probabilities. For M2(IV) sites, the second and third water molecules form strong H-bonds with chemisorbed water and framework-O atoms and promote the interaction of chemisorbed water. The adsorption energies of the second water, irrelevant of the identity of M(IV) ions, are comparable to, if not larger than, those of chemisorbed water, while those of the third and fourth water molecules, especially the latter, show reduction.

Results of CHA zeolites, where water can approach M(IV) sites from three directions, are similar to those of BEA zeolites: Sn(IV) active sites are hexa-coordinated while Ti(IV) active sites are penta-coordinated. Sn(IV) active sites in FER zeolite adopt the hexa-coordination mode as in the condition of other zeolites, while it is surprising to find that Ti(IV) active sites are also presented as hexa-coordination. Chemisorption of the first water at M(IV) sites of FER zeolites facilitates the interaction with the second water, as verified by the dihedral analyses.

Owing to enhanced structural flexibility, Lewis acidic defects reinforce the adsorption of water and the promoting effects are more obvious during chemisorption of the second water; in addition, Ti(IV) Lewis acidic defects can be expanded to hexa-coordination, while hepta-coordinated Sn9(IV) species is somewhat inhibited. M(IV) Brϕnsted acidic defects also facilitate the interaction of the first water while present the second water from chemisorption. A second form of Brϕnsted acidic defects that has higher stability exists in Sn(IV)- rather than Ti(IV)-BEA zeolites. Two forms of Sn(IV) Brϕnsted acidic defects show divergent adsorption properties and can be inter-converted during water adsorption. Despite that, all M(IV) Brϕnsted acidic defects are hexa-coordinated, irrespective of different M(IV) ions or acidic forms, due to limited space available for the second water.

## References

[1] Corma A, García H (2003). Lewis Acids: From Conventional Homogeneous to Green Homogeneous and Heterogeneous Catalysis. Chem. Rev..

[2] Luo HY, Lewis JD, Román-Leshko Y (2016). Lewis Acid Zeolites for Biomass Conversion: Perspectives and Challenges on Reactivity, Synthesis, and Stability. Annu. Rev. Chem. Biomol. Eng..

[3] Ennaert T (2016). Potential and Challenges of Zeolite Chemistry in the Catalytic Conversion of Biomass. Chem. Soc. Rev..

[4] Taramasso, M., Perego, G. & Notari, B. *US Patent 4410501* (1983).

[5] Ratnasamy. P, Srinivas D, Knözinger H (2004). Active Sites and Reactive Intermediates in Titanium Silicate Molecular Sieves. Adv. Catal..

[6] Wu P, Tatsumi T, Komatsu T, Yashima T (2001). A Novel Titanosilicate with MWW Structure. I. Hydrothermal Synthesis, Elimination of Extraframework Titanium, and Characterizations. J. Phys. Chem. B.

[7] Wu P, Miyaji T, Liu Y, He M, Tatsumi T (2005). Synthesis of Ti-MWW by a Dry-gel Conversion Method. Catal. Today.

[8] Zhou WJ (2014). Highly Selective Liquid-phase Oxidation of Cyclohexane to KA Oil over Ti-MWW Catalyst: Evidence of Formation of Oxyl Radicals. ACS Catal..

[9] Přech J, Vitvarová D, Lupínková L, Kubů M, Čejka J (2015). Titanium Impregnated Borosilicate Zeolites for EpoxidationCatalysis. Micropor. Mesopor. Mater..

[10] Přech J, Morris RE, Čejka J (2016). Selective oxidation of bulky organic sulphides over layered titanosilicate catalysts. Catal. Sci. Technol..

[11] Corma A, Nemeth LT, Renz M, Valencia S (2001). Sn-zeolite Beta as a Heterogeneous Chemoselective Catalyst for Baeyer-Villiger Oxidations. Nature.

[12] Přech J, Carretero MA, Čejka J (2017). Baeyer-Villiger Oxidation of Cyclic Ketones by Using Tin-Silica Pillared Catalysts. Chem Cat Chem.

[13] Holm MS, Saravanamurugan S, Taarning E (2010). Conversion of Sugars to Lactic Acid Derivatives Using Heterogeneous Zeotype Catalysts. Science.

[14] Moliner M, Román-Leshkov Y, Davis ME (2010). Tin-containing Zeolites are Highly Active Catalysts for the Isomerization of Glucose in Water. Proc. Natl. Acd. Sci. USA.

[15] Dapsens PY, Mondelli C, Pérez-Ramírez J (2015). Design of Lewis-acid Centres in Zeolitic Matrices for the Conversion of Renewables. Chem. Soc. Rev..

[16] Castañeda R, Corma A, Fornés V, Rey F, Rius J (2003). Synthesis of a New Zeolite Structure ITQ-24, with Intersecting 10- and 12-Membered Ring Pores. J. Am. Chem. Soc..

[17] Roth WJ (2013). A Family of Zeolites with Controlled Pore Size Prepared Using a Top-down Method. Nature Chem..

[18] Boronat M, Corma A, Renz M, Viruela PM (2006). Predicting the Activity of Single Isolated Lewis Acid Sites in Solid Catalysts. Chem. Eur. J..

[19] Zhuang JQ (2004). *In Situ* Magnetic Resonance Investigation of Styrene Oxidation over TS-1 Zeolites. Angew. Chem. Int. Ed..

[20] Román-Leshkov Y, Moliner M, Labinger JA, Davis ME (2010). Mechanism of Glucose Isomerization Using a Solid Lewis Acid Catalyst in Water. Angew. Chem. Int. Ed..

[21] Damin A, Bordiga S, Zecchina A, Lamberti C (2002). Reactivity of Ti(IV) Sites in Ti-zeolites: An Embedded Cluster Approach. J. Chem. Phys..

[22] Damin A (2002). Effect of NH_3_ Adsorption on the Structural and Vibrational Properties of TS-1. J. Phys. Chem. B.

[23] Damin A, Bordiga S, Zecchina A, Doll K, Lamberti C (2003). Ti-chabazite as a Model System of Ti(IV) in Ti-zeolites: A Periodic Approach. J. Chem. Phys..

[24] Bonino F, Damin A, Bordiga S, Lamberti C, Zecchina A (2003). Interaction of CD_3_CN and Pyridine with the Ti(IV) Centers of TS-1 Catalysts:  a Spectroscopic and Computational Study. Langmuir.

[25] Fois E, Gamba A, Spanò E (2004). Ab Initio Molecular Dynamics Simulation of the Interaction between Water and Ti in Zeolitic Systems. J. Phys. Chem. B.

[26] Zhanpeisov NU, Anpo M (2004). Hydrogen Bonding versus Coordination of Adsorbate Molecules on Ti-Silicalites:  A Density Functional Theory Study. J. Am. Chem. Soc..

[27] Deka RC (2005). Comparison of All Sites for Ti Substitution in Zeolite TS-1 by an Accurate Embedded-Cluster Method. J. Phys. Chem. B.

[28] Shetty S, Kulkarni BS, Kanhere DG, Goursot A, Pal S (2008). A Comparative Study of Structural, Acidic and Hydrophilic Properties of Sn-BEA with Ti-BEA Using Periodic Density Functional Theory. J. Phys. Chem. B.

[29] Assary RS, Curtiss LA (2011). Theoretical Study of 1,2-Hydride Shift Associated with the Isomerization of Glyceraldehyde to Dihydroxy Acetone by Lewis Acid Active Site Models. J. Phys. Chem. A.

[30] Kulkarni BS, Krishnamurty S, Pal S (2010). Probing Lewis Acidity and Reactivity of Sn- and Ti-beta Zeolite Using Industrially Important Moieties: A Periodic Density Functional Study. J. Mol. Catal. A: Chemical.

[31] Yang G, Zhou LJ, Liu XC, Han XW, Bao XH (2011). Density Functional Calculations on the Distribution, Acidity and Catalysis of the Ti^IV^ and Ti^III^ Ions in MCM-22 Zeolite. Chem. Eur. J..

[32] Yang G, Zhou LJ, Han XW (2012). Lewis and Brönsted Acidic Sites in M^4+^-doped Zeolites (M = Ti, Zr, Ge, Sn, Pb) as Well as Interactions with Probe Molecules: A DFT Study. J. Mol. Catal. A: Chemical.

[33] Yang G, Pidko EA, Hensen EJM (2013). Structure, Stability, and Lewis Acidity of Mono and Double Ti, Zr, and Sn Framework Substitutions in BEA Zeolites: A Periodic Density Functional Theory Study. J. Phys. Chem. C.

[34] Yang G, Zhou LJ (2014). Zwitterionic versus Canonical Amino Acids over the Various Defects in Zeolites: A two-layer ONIOM Calculation. Sci. Rep..

[35] Montejo-Valencia BD, Curet-Arana MC (2015). DFT Study of the Lewis Acidities and Relative Hydrothermal Stabilities of BEC and BEA Zeolites Substituted with Ti, Sn, and Ge. J. Phys. Chem. C.

[36] Li HC (2015). Structural Stability and Lewis Acidity of Tetravalent Ti, Sn, or Zr-linked Interlayer-expanded Zeolite COE-4: A DFT Study. Micropor. Mesopor. Mater..

[37] Li YP, Gomes J, Sharada SM, Bell AT, Head-Gordon M (2015). Improved Force-Field Parameters for QM/MM Simulations of the Energies of Adsorption for Molecules in Zeolites and a Free Rotor Correction to the Rigid Rotor Harmonic Oscillator Model for Adsorption Enthalpies. J. Phys. Chem. C.

[38] Yang G, Li X, Zhou LJ (2016). Adsorption of Fructose in Sn-BEA Zeolite from Periodic Density Functional Calculations. RSC Adv..

[39] Yang G, Zhu C, Zhou LJ (2016). Adsorption of Glucose within M(IV)-incorporated Zeolites: Insights from Periodic Density Functional Theory Calculations. ChemistrySelect.

[40] Han LN (2017). Density Functional Theory Investigations into the Structures and Acidity Properties of Ti-doped SSZ-13 Zeolite. Micropor. Mesopor. Mater..

[41] Karlsen E, Schöffel K (1996). Titanium-silicalite Catalyzed Epoxidation of Ethylene with Hydrogen Peroxide. A Theoretical Study. Catal. Today.

[42] Tozzola G (1998). On the Structure of the Active Site of Ti-Silicalite in Reactions with Hydrogen Peroxide: A Vibrational and Computational Study. J. Catal..

[43] Sinclair PE, Catlow CRA (1999). Quantum Chemical Study of the Mechanism of Partial Oxidation Reactivity in Titanosilicate Catalysts: Active Site Formation, Oxygen Transfer, and Catalyst Deactivation. J. Phys. Chem. B.

[44] Bonoldi L (2002). An ESR Study of Titanium-silicalite in Presence of H_2_O_2_. Spectrochim. Acta Part A.

[45] Limtrakul J, Inntam C, Truong TN (2004). Density Functional Theory Study of the Ethylene Epoxidation over Ti-substituted Silicalite (TS-1). J. Mol. Catal. A: Chemical.

[46] Spano E, Tabacchi G, Gamba A, Fois E (2006). On the Role of Ti(IV) as a Lewis Acid in the Chemistry of Titanium Zeolites: Formation, Structure, Reactivity, and Aging of Ti-Peroxo Oxidizing Intermediates. A First Principles Study. J. Phys. Chem. B.

[47] Wells DH, Joshi AM, Delgass WN, Thomson KT (2006). A Quantum Chemical Study of Comparison of Various Propylene Epoxidation Mechanisms Using H_2_O_2_ and TS-1 Catalyst. J. Phys. Chem. B.

[48] Wells DH, Delgass WN, Thomson KT (2004). Evidence of Defect-promoted Reactivity for Epoxidation of Propylene in Titanosilicate (TS-1) Catalysts:  A DFT Study. J. Am. Chem. Soc..

[49] Yang G (2008). A Joint Experimental-Theoretical Study on Trimethlyphophine Adsorption on the Lewis Acidic Sites Present in TS-1 Zeolite. J. Mol. Struct..

[50] Yuan SP (2011). Location of Si Vacancies and [Ti(OSi)_4_] and [Ti(OSi)_3_OH] Sites in the MFI Framework: A Large Cluster and Full Ab Initio Study. J. Phys. Chem. A.

[51] Yakimov AV, Kolyagin YG, Tolborg S, Vennestrøm PNR, Ivanova (2016). Irina I. ^119^Sn MAS NMR Study of the Interaction of Probe Molecules with Sn-BEA: The Origin of Penta- and Hexacoordinated Tin Formation. J. Phys. Chem. C.

[52] Sushkevich VL, Ivanova II, Yakimov AV (2017). Revisiting Acidity of SnBEA Catalysts by Combined Application of FTIR Spectroscopy of Different Probe Molecules. J. Phys. Chem. C.

[53] Montejo-Valencia BD, Salcedo-Pérez JL, Curet-Arana MC (2016). DFT Study of Closed and Open Sites of BEA, FAU, MFI, and BEC Zeolites Substituted with Tin and Titanium. J. Phys. Chem. C.

[54] Bermejo-Deval R (2012). Metalloenzyme-like Catalyzed Isomerizations of Sugars by Lewis AcidZeolites. Proc. Natl. Acd. Sci. USA.

[55] Yang G, Pidko EA, Hensen EJM (2013). The Mechanism of Glucose Isomerization to Fructose over Sn-BEA Zeolite: A Periodic Density Functional Theory Study. ChemSusChem.

[56] Rai N, Caratzoulas S, Vlachos DG (2013). Role of Silanol Group in Sn-beta Zeolite for Glucose Isomerization and Epimerization Reactions. ACS Catal..

[57] Li YP, Head-Gordon M, Bell AT (2014). Analysis of the Reaction Mechanism and Catalytic Activity of Metal-substituted Beta Zeolite for the Isomerization of Glucose to Fructose. ACS Catal..

[58] Li GN, Pidko EA, Hensen EJM (2014). Synergy between Lewis Acid Sites and Hydroxyl Groups for the Isomerization of Glucose to Fructose over Sn-containing Zeolites: A TheoreticalPerspective. Catal. Sci. Technol..

[59] Chethana BK, Mushrif SH (2005). Brønsted and Lewis Acid Sites of Sn-beta Zeolite, in Combination with the Borate Salt, Catalyze the Epimerization of Glucose: A Density Functional Theory Study. J. Catal..

[60] Christianson JR, Caratzoulas S, Vlachos DG (2015). Computational Insight into the Effect of Sn-beta Na Exchange and Solvent on Glucose Isomerization and Epimerization. ACS Catal..

[61] Wolf P (2014). NMR Signatures of the Active Sites in Sn-β Zeolite. Angew. Chem. Int. Ed..

[62] Gallo E (2013). Preference towards Five-coordination in Ti Silicalite-1 upon Molecular Adsorption. ChemPhysChem.

[63] Baerlocher, C. & McCusker, L. B. Database of Zeolite Structures. http://www.iza-structure.org/databases/ (Accessed on July 8^th^, 2015).

[64] Yang G (2008). Acidity and Defect Sites in Titanium Silicalite Catalyst. Appl. Catal. A: General.

[65] Kresse G, Furthmuller J (1996). Efficiency of Ab-initio Total Energy Calculations for Metals and Semiconductors Using a Plane-wave Basis Set. Comput. Mater. Sci..

[66] Perdew JP, Burke K, Ernzerhof M (1996). Generalized Gradient Approximation Made Simple. Phys. Rev. Lett..

[67] Grimme S (2006). Semi-empirical GGA-type Density Functional Constructed with a Long-range Dispersion Correction. J. Comput. Chem..

[68] Jain A (2011). High-throughput Infrastructure for Density Functional Theory Calculations. Comput. Mater. Sci..

[69] Opanasenko MV, Roth WJ, Čejka J (2016). Two-dimensional zeolites in catalysis: current status and perspectives. Catal. Sci. Technol..

[70] Chandrasekhar V, Nagendran S, Baskar V (2002). Organotin Assemblies Containing Sn-O Bonds. Coordin. Chem. Rev..

